# Work‐related dysphonia in subjects with occupational asthma is associated with neutrophilic airway inflammation

**DOI:** 10.1002/clt2.12218

**Published:** 2023-05-01

**Authors:** Nicolas Migueres, Olivier Vandenplas, Jolanta Walusiak‐Skorupa, Xavier Munoz, Hille Suojalehto, Vera van Kampen, Paola Mason, Santiago Quirce, Frédéric de Blay, European network for the PHenotyping of OCcupational ASthma (E‐PHOCAS)

**Affiliations:** ^1^ Department of Chest Diseases Division of Pulmonology University Hospital of Strasbourg and Fédération de Médecine translationnelle Strasbourg University Strasbourg France; ^2^ UMR 7357 Laboratoire des sciences de l'ingénieur de l'informatique et de l'imagerie ICUBE Strasbourg France; ^3^ Service de Pneumologie Centre Hospitalier Universitaire UCL Namur Université Catholique de Louvain Yvoir Belgium; ^4^ Department of Occupational Diseases and Environmental Health Nofer Institute of Occupational Medicine Lodz Poland; ^5^ Servei Pneumologia Hospital Vall d'Hebron Universitat Autonoma de Barcelona and CIBER de Enfermedades Respiratorias (CIBERES) Barcelona Spain; ^6^ Occcupational Health Finnish Institute of Occupational Health Helsinki Finland; ^7^ Institute for Prevention and Occupational Medicine of the German Social Accident Insurance (IPA) Ruhr University Bochum Germany; ^8^ Department of Cardiac‐Thoracic‐Vascular Sciences and Public Health University of Padova Padova Italy; ^9^ Department of Allergy La Paz University Hospital IdiPAZ and CIBER de Enfermedades Respiratorias (CIBERES) Madrid Spain

**Keywords:** dysphonia, neutrophilic inflammation, occupational asthma

To the editor,

Vertigan et al.^1^ recently highlighted the comorbid association between asthma and laryngeal dysfunction, although the pathophysiological mechanisms underlying this complex association remain largely uncertain.^2^ It is widely acknowledged that laryngeal dysfunction, including vocal cord dysfunction, can be triggered by external stimuli, such exercise, strong odors and irritant exposures.^2^ In this regard, workplace exposure to respiratory irritants has been reported as an important cause of the “work‐related irritable larynx syndrome”.^3^

We sought to assess the clinical characteristics and airway inflammatory processes associated with work‐related dysphonia in a cohort of subjects with sensitizer‐induced occupational asthma (OA) ascertained by a positive specific inhalation challenge (SIC). This retrospective study included 341 subjects identified among the multicenter European network for the PHenotyping of OCcupational ASthma (E‐PHOCAS)^4^ who met the following eligibility criteria: (1) complete information on variables addressing asthma severity and control while exposed at work; (2) available information on self‐reported dysphonia (i.e. hoarseness or loss of voice) at work; and (3) assessment of induced sputum cell counts at the time of the SIC procedure.

Forty‐nine (14.4%) subjects experienced dysphonia while exposed at their workplace. The baseline clinical features and sputum cell counts of the subjects with and without dysphonia as well as the univariate associations with dysphonia are detailed in Table 1. A multivariable logistic regression analysis was conducted in order to identify the clinical and inflammatory characteristics that were associated with work‐related dysphonia. The independent variables incorporated into these regression models included gender; sinusitis; high‐level treatment at work (i.e., Global Initiative for Asthma treatment step four‐fifths); poor asthma control at work (i.e., need for an inhaled short‐acting β_2_‐agonist once or more a day); OA caused by a low‐ versus a high‐molecular‐weight agent; as well as eosinophil and neutrophil sputum cell counts (expressed as % of total nonsquamous cells; Table 2).The multivariate logistic regression analysis revealed that female gender (odds ratio [OR], 2.04; 95% confidence interval [CI], 1.06–3.92; *p* = 0.031) and a higher sputum neutrophil count (OR for each 5%‐increase in neutrophil count, 1.09; 95% CI, 1.01–1.18; *p* = 0.025) were significantly associated with a higher likelihood of work‐related dysphonia (Table 2). There was an association of borderline significance between dysphonia and high‐level treatment (OR, 1.97; 95% CI, 0.97–3.95; *p* = 0.057). Dysphonia showed a negative association with increased sputum eosinophil counts (OR, 0.41; 95% CI, 0.19–0.83; *p* = 0.017).

**TABLE 1:** Univariate associations with self‐reported dysphonia at work

Characteristics	Missing values	Subjects without dysphonia at work (*n* = 292)	Subjects with dysphonia at work (*n* = 49)	OR (95% CI)	*p* value
Age, year^a^	0	43 (34–51)	42 (38–52)	1.02 (0.99–1.05)	0.279
Sex, female	0	97 (33.2)	23 (46.9)	**1.78 (0.96–3.28)**	**0.065**
Body mass index ≥30 kg/m^2^ ^a^	0	82 (28.1)	15 (30.6)	1.13 (0.57–2.15)	0.717
Ex‐smokers	0	83 (28.4)	10 (20.4)	0.66 (0.29–1.38)	0.286
Current smokers	0	62 (21.2)	12 (24.5)	1.05 (0.49–2.17)	0.890
Atopy^b^	4/0	146 (50.7)	28 (57.1)	1.30 (0.71–2.41)	0.405
Chronic rhinosinusitis	2/0	21 (7.2)	8 (16.3)	**2.50 (0.99–5.83)**	**0.041**
Exposure before symptom onset, months^a^	2/0	108 (48–204)	150 (21–230)	1.00 (1.00–1.00)	0.218
Duration of asthma symptoms at work, months^a^	3/0	36 (16–84)	33 (21–68)	1.00 (0.99–1.00)	0.522
Type of causal agent, LMW	0	191 (65.4)	25 (51.0)	**1.81 (0.98–3.33)**	**0.057**
Asthma treatment at work					
Daily dose of ICS, μg^a^ ^,^ ^c^	0	500 (0–1000)	500 (0–1000)	1.00 (1.00–1.00)	0.827
High level treatment^d^	0	19 (6.5)	6 (12.2)	**2.12 (1.14–3.94)**	**0.017**
Poor asthma control while at work^e^	0	74 (25.3)	21 (42.9)	**2.21 (1.17–4.11)**	**0.013**
≥2 exacerbations last 12 months at work	0	26 (8.9)	1 (2.0)	0.21 (0.01–1.04)	0.134
Baseline spirometry					
FVC, % pred^a^	0	101 (90–110)	103 (94–110)	1.01 (0.99–1.03)	0.610
FEV_1_, % pred^a^	0	90 (79–98)	91 (78–98)	1.01 (0.98–1.03)	0.602
FEV_1_/FVC, %^a^	0	74 (67–80)	75 (67–78)	1.00 (0.97–1.03)	0.981
Airflow obstruction^f^	0	56 (19.2)	13 (26.5)	1.52 (0.73–3.00)	0.238
Baseline NSBH	22/0				
Absent		56 (20.7)	11 (22.4)	1.11 (0.51–2.24)	0.787
Mild		139 (51.5)	27 (55.1)	1.16 (0.63–2.15)	0.641
Moderate/severe		75 (27.8)	11 (22.4)	0.75 (0.35–1.50)	0.440
Blood eosinophils, cells/μl^a^	58/10	280 (199–400)	249 (140–390)	1.00 (1.00–1.00)	0.335
Baseline FeNO, ppb^a^	184/10	22 (12–41)	22 (10–28)	**0.98 (0.96–1.00)**	**0.049**
Baseline sputum eosinophils					
%^a^	0	2.0 (1.0–6.0)	1.2 (0.2–2.5)	**0.87 (0.77–0.95)**	**0.011**
≥3%		125 (42.8)	12 (24.5)	**0.43 (0.21–0.84)**	**0.018**
Baseline sputum neutrophils					
%^a^	0	51.0 (36.0–70.0)	60.0 (48.2–78.5)	**1.02 (1.00–1.03)**	**0.017**
≥76%		57 (19.5)	15 (30.6)	**1.82 (0.91–3.52)**	**0.081**

*Note*: Data are presented as *n* (% of available data) unless otherwise specified. Bold indicates variable with univariate association demonstrating a *p* value under 0.1.

Abbreviations: FeNO, fractional exhaled nitric oxide; FEV_1_, forced expiratory volume in one‐second; FVC, forced vital capacity; ICS, inhaled corticosteroid; LMW, low‐molecular‐weight; NSBH, nonspecific bronchial hyperresponsiveness; SIC, specific inhalation challenge.

^a^ Median value with interquartile range (IQR) within parentheses.

^b^ Atopy defined by the presence of at least one positive skin prick test result to common allergens.

^c^ Daily dose of inhaled corticosteroid expressed as beclomethasone dipropionate equivalent.

^d^ High‐level treatment defined as treatment step 4 or 5 of the Global Initiative for Asthma (http://www.ginasthma.org).

^e^ Poor asthma control at work is defined as the use of SABA more than once a day.

^f^ Airflow obstruction defined by an FEV_1_ <80% predicted and an FEV_1_/FVC ratio <70%.

**TABLE 2:** Logistic multivariate model for dysphonia at work

	Dysphonia at work (*n* = 49/341)		
Independent variables	OR	(95% CI)	*p* value
Sex, female	2.04	(1.06–3.92)	**0.031**
Chronic rhinosinusitis			
Poor asthma control while at work^a^	1.84	(0.91–3.71)	0.087
Type of causal agent, LMW			
High level treatment^b^	1.97	(0.97–3.95)	0.057
Eosinophil sputum cell counts ≥3%	0.41	(0.19–0.83)	**0.017**
Neutrophil sputum cell counts, 5% increase	1.05	(1.03–1.07)	**<0.001**

*Note*: The model included 338 patients, selection of variables was realized by a stepwise procedure based on Akaike information criterion. Bold indicates variable associated with a statistical significance, demonstrating a *p* value under 0.05.

Abbreviation: LMW, low‐molecular‐weight.

^a^ Poor asthma control at work is defined as the use of SABA more than once a day.

^b^ High‐level treatment defined according to GINA as treatment step 4 or 5.

Dysphonia is a main symptom of worked‐associated irritable larynx syndrome (WILS) which has been defined as neuronal sensitization by a workplace trigger bringing about laryngeal dysfunction.^3^ As recently described, neutrophil inflammation can regulate sensory neuron function, especially in chronic pain.^5^

To our knowledge, our study is the first to describe a relationship between neutrophilic inflammation and work related dysphonia.

We acknowledge the limitations inherent to the retrospective cross‐sectional design of this study. The presence of dysphonia was not objectively documented through direct visualization of inappropriate laryngeal movement. In addition, dysphonia was not assessed during the SIC procedure implying that it was not possible to ascertain that the agent inducing the positive SIC response was also the cause of dysphonia at work.

Despite their inherent limitations, our findings suggest that airway neutrophilic inflammation could be involved in the development of work‐related laryngeal dysfunction. This study highlights the need for further prospective studies using validated questionnaires, laryngoscopy, and induced sputum analysis in order to explore the association between laryngeal dysfunction and neutrophilic airway inflammation.

## AUTHOR CONTRIBUTIONS

All E‐PHOCAS investigators contributed to the conception and design of the study. All authors contributed to the acquisition, analysis and interpretation of the data. They provided input into the drafting of the manuscript, critical feedback, and final approval for submission of the manuscript for publication. NM is the guarantor of the final content of the manuscript.

## CONFLICT OF INTEREST

The authors have no conflicts of interest to declare.

## FUNDING INFORMATION

Association d'Aide aux Insuffisants Respiratoires d'Alsace (ADIRAL); Fondation Mont‐Godinne

## Supporting information

Supporting Information S1

## Data Availability

Research data are not shared.
