# Supplementary material for: Work‐related dysphonia in subjects with occupational asthma is associated with neutrophilic airway inflammation
Source: Clin Transl Allergy. 2023 May 1;13(5):e12218. doi: 10.1002/clt2.12218 (PMC10151386; doi:10.1002/clt2.12218)
Supplement: Supplementary file 1 — Supporting Information S1 [file CLT2-13-e12218-s001.docx]

**SUPPLEMENTARY MATERIALS**

**List of investigators of the European network for the PHenotyping of OCcupational ASthma (E-PHOCAS)**

- Olivier Vandenplas, Catherine Rifflart (Department of Chest Medicine, Centre Hospitalier Universitaire UCL Namur, Université Catholique de Louvain, Yvoir, Belgium);
- Pavlina Klusackova (Department of Occupational Medicine, General University Hospital, 1st Faculty of Medicine, Charles University, Prague, Czech Republic);
- David Sherson, Jesper Baelum (Department of Pulmonary Medicine and Occupational Medicine, Odense University Hospital, Odense, Denmark);
- Hille Suojalehto, Katri Suuronen, Irmeli Lindström, Pirjo Hölttä (Occcupational Health, Finnish Institute of Occupational Health, Helsinki, Finland);
- Paula Kauppi (Department of Allergy, Skin and Allergy Hospital, Helsinki University Central Hospital, Helsinki, Helsinki, Finland);
- Frédéric de Blay, Nicolas Migueres (Division of Pulmonology, Department of Chest Diseases, University Hospital of Strasbourg, Fédération de Médecine translationnelle, Strasbourg University Strasbourg, France);
- Rolf Merget, Vera van Kampen (Institute for Prevention and Occupational Medicine of the German Social Accident Insurance [IPA], Ruhr University, Bochum, Germany);
- Alexandra M Preisser (Institute for Occupational and Maritime Medicine, University Medical Center Hamburg-Eppendorf, Hamburg, Germany);
- Piero Maestrelli, Paola Mason (Department of Cardiac-Thoracic-Vascular Sciences and Public Health, University of Padova, Padova, Italy);
- Gianna Moscato, Patrizia Pignatti (Department of Public Health, Experimental and Forensic Medicine, Specialization School in Occupational Medicine, University of Pavia, Pavia, Italy);
- Pierluigi Paggiaro, Donatella Talini (Cardio-Thoracic and Vascular Department, University of Pisa, Pisa, Italy);
- Marco dell’Omo, Ilenia Foletti (Department of Medicine, Section of Occupational Medicine, Respiratory Diseases and Occupational and Environmental Toxicology, University of Perugia, Italy);
- Cecilie Svanes, Jorunn Kirkeleit, Thomas Blix Grydeland (Department of Occupational Medicine, Haukeland University Hospital, Bergen, Norway);
- Jolanta Walusiak-Skorupa, Marta Wiszniewska, Agnieszka Lipińska-Ojrzanowska (Department of Occupational Diseases and Environmental Health, Nofer Institute of Occupational Medicine, Lodz, Poland);
- Xavier Munoz, Christian Romero-Mesones (Servei Pneumologia, Hospital Vall d’Hebron, Universitat Autonoma de Barcelona and CIBER de Enfermedades Respiratorias [CIBERES], Barcelona, Spain);
- Joaquin Sastre, Mar Fernandez-Nieto (Department of Allergy, Fundacion Jimenez Dıaz, Universidad Autonoma de Madrid and CIBER de Enfermedades Respiratorias [CIBERES], Madrid, Spain);
- Santiago Quirce, David Loli (Department of Allergy, La Paz University Hospital, IdiPAZ and CIBER de Enfermedades Respiratorias [CIBERES], Madrid, Spain;
- Paul Cullinan, Julie Cannon (Department of Occupational and Environmental Medicine, Royal Brompton Hospital and Imperial College [NHLI], Royal Brompton and Harefield NHS Foundation Trust, London, UK);
- Sherwood Burge, Vicky Moore (Occupational Lung Disease Unit, Birmingham Heartlands Hospital, Birmingham, UK);
- Jennifer Hoyle (Department of Respiratory Medicine, North Manchester General Hospital, Manchester, UK).

**METHODS**

**Cohort Recruitment**

This retrospective cross-sectional study was conducted in the context of the multicenter cohort of the European network for the PHenotyping of OCcupational Asthma (E-PHOCAS)(1–5).This cohort aimed at recruiting all patients with occupational asthma (OA) due to various occupational agents documented by a specific inhalation challenge (SIC) completed in 20 European centers between 2006 and 2018. Overall, these centers reported 1,518 subjects with OA due to various occupational agents. From these initially reported subjects who fulfilled the criteria for a positive SIC (see below), 221 subjects were excluded because of incomplete information pertaining to key asthma outcomes (i.e., detailed asthma medications while exposed at work and at the time of the SIC and number of severe asthma exacerbations over the last 12 months at work). Eligible subjects for this analysis were those with complete information on variables addressing asthma severity and control while exposed at work; available information on self-reported dysphonia (i.e. hoarseness or loss of voice) at work; and assessment of induced sputum cell counts at the time of the SIC procedure.

Eight of the 20 participating centers used the induced sputum technique for periods ranging from one to 13 years (median: 6 years) during the 2006-2018 study period. Over the years during which induced sputum was performed, a total of 651 subjects underwent an SIC procedure in these eight centers. Of these, 361 (55.4%) subjects completed at least one sputum induction while 290 subjects had no sputum data. Three hundred and forty one subjects had complete data on eosinophil and neutrophil percentages at the time of the challenge without missing information about self-reported dysphonia at work and variables addressing asthma severity and control while exposed at work.

**Data Collection**

Anonymized information on demographic, clinical, occupational, and physiological characteristics of the subjects collected at the time of the diagnostic evaluation was entered in a standardized spreadsheet in each participating center. These local databases were then checked for inconsistencies and missing data by three investigators (CR, NM, and OV), pooled together and centralized at the Strasbourg University.

The database gathered information on the following items: 1) causal agent and job; 2) demographic and clinical characteristics; 3) timing of work-related respiratory symptoms in relation to occupational exposure; 4) co-existing conditions (i.e. work-related rhinitis, dysphonia, contact urticaria and/or dermatitis, and chronic rhinosinusitis); 5) asthma medication, including frequency of short-acting β_2_-agonist use, while exposed at work and at the time of the SIC procedure; and 6) severe asthma exacerbations during the last 12 months at work.

Since most of the participating centers failed to use validated instruments for the assessment of asthma control throughout the study period, “poor symptom control” was defined by the need for an inhaled short-acting β_2_-agonist (SABA) once or more a day as proposed in the recommendations of the American Thoracic Society (ATS) issued in 2000 (6) .

**Assessment of Nonspecific Bronchial Hyperresponsiveness**

The level of nonspecific bronchial hyperresponsiveness (NSBH) was expressed as the concentration or dose of the pharmacological agent inducing a 15% or 20 % fall in FEV_1_ (PC/PD_15-20%_) according to the bronchoprovocation method used in each center. Since participating centers used different methods, the level of NSBH was only categorized as “absent”, “mild”, or “moderate-to-severe” based on the available recommendations (7–9) or a consensus Delphi approach among investigators (1) . The bronchoprovocation methods and threshold values used for defining the level of NSBH in the 341 subjects included in this analysis are detailed in Table S1.

**Methodology of Specific Inhalation Challenges**

In order to evaluate the compliance with international recommendations on SIC with occupational agents (10), the investigators completed a questionnaire on the following items prior to participating in the E-PHOCAS cohort: 1) absence of respiratory tract infection or asthma exacerbation within the previous 4 weeks; 2) duration of ICS withdrawal before the SIC procedure; 3) performance of a control (placebo) test on a separate day before challenging the subjects with occupational agents; 4) lower limit of FEV_1_ value considered a contra-indication for performing a SIC procedure 5) method used for delivering challenge exposures with workplace agents (i.e., “realistic” challenge or inhalation of an “allergen extract”; and 6) functional monitoring of at least 6 hours after the end of challenge exposure.

All participating centers conformed with safety and reliability requirements. The lower limit of FEV_1_ was 70% of the predicted value in three centers, 65% in one center; 60% in four centers. In all centers, ICS were withheld 2 or 3 days before the SIC procedure.

The results of the SICs were interpreted *a posteriori* according to standardized criteria. A positive SIC result was defined by either a ≥15% fall in FEV_1_ at any time during the post-challenge monitoring or a twofold or greater increase in the post-challenge level of NSBH (i.e., a pre/post PC/PD_15-20%_ ratio ≥2) in the absence of a ≥15% fall in FEV_1_ (10) .

**Sputum Induction and Processing**

The eight participating centers completed a detailed questionnaire pertaining to the method used for the induction and analysis of sputum samples. Sputum was induced through different methods, including the inhalation of nebulized isotonic saline (n=1), a single concentration of hypertonic solutions (i.e., 3%; n=1) or increasing concentrations of hypertonic solutions ranging (i.e., 3%, 4%, and 5%; n=7) for a maximum cumulative duration of 15 to 40 minutes (11). The processing of sputum samples was carried out either by selecting viscid portions from the expectorate (3 centers) (12) or using the whole expectorate (5 centers) (13). Homogenization of the sample was achieved by adding dithiothreitol (0.1%). All centers applied quality criteria based on the cell viability (i.e. at least 40%) and the level of contamination by squamous cells (11). The accepted squamous cell contamination was <20% in five centers, <30% in one center, and <50% in two centers. The differential cell count was determined by counting a minimum of 400 nonsquamous cells. Sputum eosinophil and neutrophil counts collected at baseline and 24 hours after the challenge exposure were expressed as a percentage of nonsquamous cells. Available information indicates that using viscid portions from the expectorate or the whole expectorate as well as different nebulizers and saline concentrations does not significantly affect differential sputum cell counts (11,14) .

**Fractional Exhaled Nitric Oxide**

The FeNO level was measured at baseline and 24 hours post-SIC at a flow rate of 50 ml/s using different devices in compliance with the recommendations of the European Respiratory Society and the American Thoracic Society (15).

**Table S1. Methods used for measuring and grading the level of nonspecific bronchial hyperresponsiveness**

| **Method (pharmacological agent)** | **No. of centers (subjects)*** | **Threshold values for nonspecific bronchial hyperresponsiveness** | | |
| --- | --- | --- | --- | --- |
|  |  | **Moderate-to-severe** | **Mild** | **Absent** |
| Tidal breath method  (histamine/methacholine) [9, 10] | 2 (212) | PC_20_ <1 mg/ml | PC_20_:1-16 mg/ml | PC_20_ >16 mg/ml |
| Five-breath dosimeter method  (methacholine) [9, 10] | 5 (43) | PD_20_ <0.1 mg  PC_20_ <1 mg/ml | PD_20_: 0.1-1.5 mg  PC_20_:1-16 mg/ml | PD_20_ >1.5 mg  PC_20_ >16 mg/ml |
| Rapid dosimeter method  (histamine) [11] | 1 (17) | PD_15_ <0.4 mg | PD_15_: 0.4-1.6 mg | PD_15_ >1.6 mg |
| Reservoir bag dosimeter method  (methacholine) [21] | 1 (4) | PD_20_ or PD_100_ sRt <0.1 mg | PD_20_ or PD_100_ sRt: 0.1-0.3 mg | PD_20_ or PD_100_ sRt >0.3 mg |

Legend: *PC_/_PD_15-20_*, provocative concentration/dose of pharmacological agent inducing a 15 or 20% fall in FEV_1_; *PD_100_ sRt*: provocative concentration of pharmacological agent inducing a doubling of specific airway resistance (sRt).

**REFERENCES**

1. Vandenplas O, Godet J, Hurdubaea L, Rifflart C, Suojalehto H, Wiszniewska M et al. Are high- and low-molecular-weight sensitizing agents associated with different clinical phenotypes of occupational asthma? *Allergy* 2019;**74**:261–272.

2. Vandenplas O, Godet J, Hurdubaea L, Rifflart C, Suojalehto H, Walusiak-Skorupa J et al. Severe Occupational Asthma: Insights From a Multicenter European Cohort. *The Journal of Allergy and Clinical Immunology: In Practice* 2019;**7**:2309-2318.e4.

3. Suojalehto H, Suuronen K, Cullinan P, Lindström I, Sastre J, Walusiak-Skorupa J et al. Phenotyping Occupational Asthma Caused by Acrylates in a Multicenter Cohort Study. *The Journal of Allergy and Clinical Immunology: In Practice* 2019;:S2213219819309080.

4. Wiszniewska M, Dellis P, van Kampen V, Suojalehto H, Munoz X, Walusiak-Skorupa J et al. Characterization of Occupational Eosinophilic Bronchitis in a Multicenter Cohort of Subjects with Work-Related Asthma Symptoms. *The Journal of Allergy and Clinical Immunology: In Practice* 2021;**9**:937-944.e4.

5. Migueres N, Debaille C, Walusiak-Skorupa J, Lipińska-Ojrzanowska A, Munoz X, van Kampen V et al. Occupational Asthma Caused by Quaternary Ammonium Compounds: A Multicenter Cohort Study. *J Allergy Clin Immunol Pract* 2021;:S2213-2198(21)00503-1.

6. Proceedings of the ATS Workshop on Refractory Asthma: Current Understanding, Recommendations, and Unanswered Questions. *Am J Respir Crit Care Med* 2000;**162**:2341–2351.

7. Sterk PJ, Fabbri LM, Quanjer PH, Cockcroft DW, O’Byrne PM, Anderson SD et al. Airway responsiveness. Standardized challenge testing with pharmacological, physical and sensitizing stimuli in adults. Report Working Party Standardization of Lung Function Tests, European Community for Steel and Coal. Official Statement of the European Respiratory Society. *Eur Respir J Suppl* 1993;**16**:53–83.

8. Crapo RO, Casaburi R, Coates AL, Enright PL, Hankinson JL, Irvin CG et al. Guidelines for methacholine and exercise challenge testing-1999. This official statement of the American Thoracic Society was adopted by the ATS Board of Directors, July 1999. *Am J Respir Crit Care Med* 2000;**161**:309–329.

9. Sovijärvi AR, Malmberg LP, Reinikainen K, Rytilä P, Poppius H. A rapid dosimetric method with controlled tidal breathing for histamine challenge. Repeatability and distribution of bronchial reactivity in a clinical material. *Chest* 1993;**104**:164–170.

10. Vandenplas O, Suojalehto H, Aasen TB, Baur X, Burge PS, de Blay F et al. Specific inhalation challenge in the diagnosis of occupational asthma: consensus statement. *European Respiratory Journal* 2014;**43**:1573–1587.

11. Efthimiadis A, Spanevello A, Hamid Q, Kelly MM, Linden M, Louis R et al. Methods of sputum processing for cell counts, immunocytochemistry and in situ hybridisation. *Eur Respir J Suppl* 2002;**37**:19s–23s.

12. Pin I, Gibson PG, Kolendowicz R, Girgis-Gabardo A, Denburg JA, Hargreave FE et al. Use of induced sputum cell counts to investigate airway inflammation in asthma. *Thorax* 1992;**47**:25–29.

13. Fahy JV, Liu J, Wong H, Boushey HA. Cellular and biochemical analysis of induced sputum from asthmatic and from healthy subjects. *Am Rev Respir Dis* 1993;**147**:1126–1131.

14. Pizzichini E, Pizzichini MM, Efthimiadis A, Evans S, Morris MM, Squillace D et al. Indices of airway inflammation in induced sputum: reproducibility and validity of cell and fluid-phase measurements. *Am J Respir Crit Care Med* 1996;**154**:308–317.

15. Dweik RA, Boggs PB, Erzurum SC, Irvin CG, Leigh MW, Lundberg JO et al. An official ATS clinical practice guideline: interpretation of exhaled nitric oxide levels (FENO) for clinical applications. *Am J Respir Crit Care Med* 2011;**184**:602–615.
